# Application of a scheimpflug-based biomechanical analyser and tomography in the early detection of subclinical keratoconus in chinese patients

**DOI:** 10.1186/s12886-021-02102-2

**Published:** 2021-09-20

**Authors:** Yan Liu, Yu Zhang, Yueguo Chen

**Affiliations:** 1 Department of Ophthalmology, Peking University Third Hospital, Beijing, China; 2 Beijing Key Laboratory of Restoration of Damaged Ocular Nerves, Peking University Third Hospital, 49 North Garden Road, Haidian District, 100191 Beijing, China

**Keywords:** keratoconus, forme frusta keratoconus, corneal biomechanics, corneal tomography

## Abstract

**Background:**

In vivo corneal biomechanics evaluation has been used to help screen early keratoconus in recent years. This study is to evaluate the value of a Scheimpflug-based biomechanical analyser combined with tomography in detecting subclinical keratoconus by distinguishing normal eyes from frank keratoconus (KC) and forme frusta keratoconus (FFKC) eyes in Chinese patients.

**Methods:**

Study design: diagnostic test. This study included 31 bilateral frank keratoconus patients, 27 unilateral clinically manifesting keratoconus patients with very asymmetric eyes, and 79 control subjects with normal corneas. Corneal morphological and biomechanical parameters were measured using a Pentacam HR and a Corvis ST (OCULUS, Wetzlar, Germany). The diagnostic ability of computed parameters reflecting corneal biomechanical and morphological traits [including the Belin-Ambrósio deviation index (BAD_D), the Corvis biomechanical index (CBI) and the tomographic and biomechanical index (TBI)] was determined using receiver operating characteristic (ROC) curve analysis and compared by the DeLong test. Additionally, the area under the curve (AUC), the best cut-off values, and the Youden index for each parameter were reported. A novel corneal stiffness parameter, the stress-strain index (SSI), was also compared between KC, FFKC and normal eyes.

**Results:**

Every morphological and biomechanical index analysed in this study was significantly different among KC, FFKC and normal eyes (*P* = 0.000). The TBI was most valuable in detecting subclinical keratoconus (FFKC eyes), with an AUC of 0.928 (*P* = 0.000), and both forms of corneal ectasia (FFKC and frank KC eyes), with an AUC of 0.966 (*P* = 0.000). The sensitivity and specificity of the TBI was 97.5 and 77.8 % in detecting FFKC and 97.5 and 89.7 % in detecting any KC, respectively, with a cut-off value of 0.375. The morphological index BAD_D and the biomechanical index CBI were also very useful in distinguishing eyes with any KC from normal eyes, with AUCs of 0.965 and 0.934, respectively. The SSI was significantly different between KC, FFKC and normal eyes (*P* = 0.000), indicating an independent decrease in corneal stiffness in KC eyes.

**Conclusions:**

The combination of a Scheimpflug-based biomechanical analyser and tomography could increase the accuracy in detecting subclinical keratoconus in Chinese patients. The TBI was the most valuable index for detecting subclinical keratoconus, with a high sensitivity and specificity. Evaluation of corneal biomechanical properties in refractive surgery candidates could be helpful for recognizing potential keratoconic eyes and increasing surgical safety.

## Introduction

Laser vision correction (LVC) surgery has gained increasing attention and become quite widespread due to the soaring prevalence of myopia in China [1, 2]. Postoperative iatrogenic corneal ectasia is a very severe surgical complication that can cause irreversible loss of corrected visual acuity [3]. Thus, it is of paramount importance to detect the predisposition to corneal ectasia to avoid potential LVC surgery complications and improve vision prognosis.

Placido disk-based topography, mainly focusing the features of corneal anterior surface, has been used as a classic method for screening corneal ectasia for many years [4]. In recent decades, Scheimpflug-based tomography has been introduced to evaluate corneal morphology, including both front and back corneal surfaces [5]. Scheimpflug-based tomography is a non-contact optical system involving a rotating Scheimpflug camera that takes up to 100 slit images of the anterior segment of the eye in less than 2 s [5]. The latest global consensus on KC in 2015 proposed that the condition initiates from the posterior surface of the cornea [6]; thus, Scheimpflug-based tomography is superior to traditional topography in detecting suspected or subclinical KC. The Pentacam HR (OCULUS Optikgeräte GmbH; Wetzlar, Germany) is a widely used Scheimpflug-based tomographic device, and the Belin-Ambrósio deviation index (BAD) is a computed index used to assess the predisposition of keratoconus using Pentacam parameters, comprising a combination of ‘D’ values using logistic regression analysis to optimize ectasia detection [7]. Different studies have found that the BAD_D is a very accurate parameter for detecting ectasia, with a relatively high sensitivity and specificity [8–10]. Although many novel instruments have been applied to detect the potential predisposition to ectasia, such predictions remain challenging for refractive surgeons. There are still sporadic reports of patients with relatively normal topography progressing to corneal ectasia after LVC surgery [11, 12]. Therefore, a new screening method is imperative to increase the diagnostic accuracy, especially for topography-normal eyes.

In recent years, in vivo corneal biomechanical assessment has emerged for detecting suspected or subclinical keratoectasia. The Corvis ST (OCULUS Optikgeräte GmbH; Wetzlar, Germany) is a recently developed non-contact biomechanical measurement device [13]. The Corvis biomechanical index (CBI) is an integration of several dynamic corneal response parameters measured by the Corvis ST, reflecting a comprehensive corneal biomechanical property [14]. Recently, Ambrósio and coworkers developed a novel combined index, the tomographic/biomechanical index (TBI), which enables the robust integration of corneal morphology from the Pentacam HR and corneal biomechanics from the Corvis ST. The TBI is calculated from the random forest method with a leave-one-out cross-validation (RF/LOOCV) model, taking both corneal morphological and biomechanical characteristics into consideration, which further improves the accuracy of mild keratoectasia detection [15].

Subclinical keratoconus recognition is a constant challenge for ophthalmologists. In some cases, subclinical keratoconus is so difficult to identify that the diagnosis can only be confirmed by follow-up sessions over years. Fortunately, there are some unilateral clinical manifestations in keratoconus patients with very asymmetric eyes (VAEs). According to the 2015 KC global consensus, “*true unilateral keratoconus does not exist*” [6]. Although the corneal topography of the other eye (the FFKC eye) is relatively normal, early corneal disease can quietly occur. Analysing the morphology and biomechanics of FFKC eyes could provide valuable information for subclinical KC diagnoses. Considering previous studies have mostly focused on a non-Chinese population [14–18], the purpose of the current study was to evaluate the accuracy of detecting frank KC and FFKC in Chinese patients by comparing the morphological and biomechanical parameters.

## Methods

### Study design

This study is a diagnostic test with retrospective design to compare instrument accuracy.

### Participants

This study enrolled 137 continuous subjects from June 2019 to June 2020 in Peking University Eye Center, Beijing, China. Written informed consent was obtained from the subjects. This study followed the tenets of the Declaration of Helsinki, and the study protocol was approved by the Medical Science Research Ethics Committee of Peking University Third Hospital.

All the participants were divided into three groups: the bilateral KC group, the very asymmetric eye (VAE) group and the control group. The clinical diagnosis of KC was based on slit-lamp findings (i.e., stromal thinning, conical protrusion of the corneal apex, Fleischer rings, Vogt striae or anterior stromal scars) and the presence of abnormal topographic patterns on the sagittal front curvature map [19], disregarding tomographic and biomechanical findings, and was confirmed by an experienced specialist majoring in corneal and LVC surgery. VAEs included the frank ectatic eyes and the fellow forme fruste keratoconus (FFKC) eyes. FFKC eyes were diagnosed by a specialist according to the following criteria [20, 21]:

1. CDVA ≥ 20/20 Snellen equivalent (≤ 0 Logarithm of the Minimum Angle of Resolution [LogMAR]).
2. Eyes with normal topography as obtained with an Allegro Topolyzer (WaveLight Technologie AG, Alcon Laboratories, Erlangen, Germany), with a KC grading of KC0.
3. A mean keratometry (K) value < 47 dioptres (D) and an inferior–superior (I-S) value ≤ 1.4 D according to the Rabinowitz and McDonnell criteria [21];
4. Pachymetry at the thinnest location > 470 μm.
5. No signs of KC under slit lamp examination, and no central/paracentral or inferior focal steepening (anterior and/or posterior) and/or corneal thinning.
6. Confirmed KC in the fellow eye.

The exclusion criteria included previous ocular surgery or trauma history, significant corneal scarring or associated ocular pathology. All participants did not wear soft contact lenses 2 weeks, rigid gas permeable (RGP) lenses 4 weeks before the examinations, otherwise they were excluded.

### Procedure

All the participants underwent basic eye examinations, including visual acuity, slit-lamp examination, indirect ophthalmoscopic fundus examination, refraction and corneal topography (WaveLight Allegro Topolyzer, Alcon Surgical). Furthermore, all eyes were examined by rotating Scheimpflug corneal tomography (Pentacam HR). Scans that were registered as “OK” or “model deviation” on the examination quality specification were included for analysis.

Corneal biometric parameters were measured using a Corvis ST II. The Corvis ST II is a novel-developed tool for measuring corneal deformation in a non-contact mode by a released air puff (60 mmHg of pressure, air puff diameter 3.05 mm). Video footage of the corneal deformation was obtained by a Scheimpflug camera angled at 45° towards the apex of the cornea. A total of approximately 140 cross-sectional images of the cornea were collected over a collimated air puff for 30 ms [15]. Biomechanical parameters were measured at the end of this process by built-in software (Version 1.4r1755). All examinations were performed by a single experienced technician in the same examining room under low light conditions to avoid bias [22, 23].

### Data collection

Corneal morphological parameters were obtained from Pentacam examination, including the simulated keratometry of the flat and steep meridians in the central 3 mm of the front and back corneal surfaces; the maximum keratometry (Kmax) of the front corneal surface; the central corneal thickness (CCT) at the apex and the thinnest point; the Ambrósio relational thickness to the horizontal profile (ARTh); the inferior-superior difference (I-S) value; and the Belin–Ambrósio enhanced ectasia total deviation (BAD_D) index.

The following biomechanical parameters were obtained from the Corvis device: SPA_1 (resultant pressure divided by the deflection amplitude at A1), integrated radius (IR, area under the inverse concave radius curve), and DA ratio_2 (the ratio between DA at the apex and the average of the DAs at 2 mm around the centre in the temporal and nasal directions). The novel parameters, the CBI (a combination of the dynamic corneal response parameters and the corneal thickness profile in the horizontal meridian) and the TBI were analysed to assess their discriminability. A new in vivo biomechanical parameter, the stress-strain index (SSI), was introduced and collected in this study [24].

In the KC group and control group, a randomly selected eye was included in the data analysis to avoid bias. In the VAE group, FFKC eyes were included in analysis. At the data analysis stage, the KC group included one eye from the bilateral KC group and the ectasia eye from the VAE group (35 eyes in total). The FFKC group included the FFKC eyes from VAE patients (22 eyes). The control group included one eye from the control group, as mentioned earlier (56 eyes).

### Statistical analysis

Data were analysed using SPSS 22 software (SPSS Inc., Chicago, IL, USA). The normality of the data distribution was assessed with the Kolmogorov-Smirnov goodness-of-fit test. Data following a normal distribution were compared by one-way analysis of variance (ANOVA); otherwise, they were compared by the non-parametric Kruskal-Wallis test between groups. The Bonferroni test and post hoc test for Kruskal-Wallis analysis were used for pairwise comparisons. Receiver operating characteristic (ROC) curves were used to illustrate the sensitivity and specificity for different cut-off points of the corneal morphological and biomechanical parameters in KC, FFKC and control eyes. Moreover, the best cut-off value, the area under the ROC curve (AUC), and the Youden index for BAD_D, CBI and TBI were determined. An AUC value of 1.0 indicates perfect discrimination, whereas values less than 0.5 show that the assessed parameter has no diagnostic ability. Pairwise comparison of the AUCs was performed using the DeLong test [25]. The sample size was calculated according to the reported sensitivity and specificity of TBI in diagnosing KC or VAEs in PASS 15.0 [15, 26]. *P* < 0.05 was considered statistically significant for all tests.

## Results

**Fig. 1:**
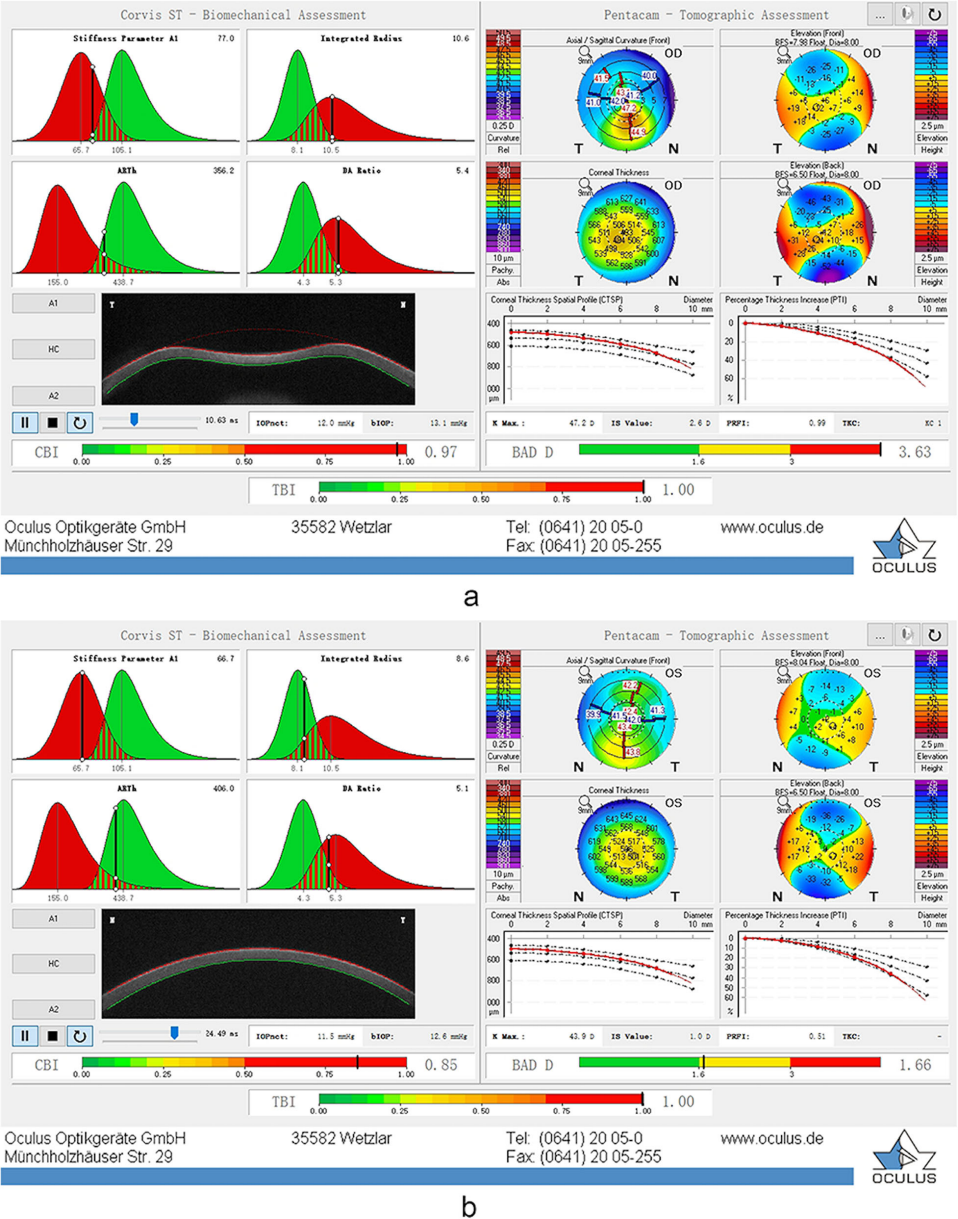
One VAE patient with corneal ectasia (**a**) in the right eye and FFKC (**b**) in the left eye

A total of 150 patients were examined for inclusion of this study, of which 13 were excluded for severe corneal scarring or RGP wearing. Among these 137 patients, 31 were diagnosed with bilateral keratoconus, 27 had unilateral frank keratoconus with very asymmetric eyes (VAEs), and 79 participants had no signs of keratoconus. The mean ages of the participants in the bilateral KC group, VAE group and control group were 23.81 ± 6.98, 22.00 ± 6.26 and 24.87 ± 7.62 years, respectively. ANOVA showed that there was no significant difference in age between the three groups (*P* = 0.203), ensuring comparability among the groups. The male/female ratios were 25/6, 16/11 and 47/32 in the respective groups (chi-squared test, *P* < 0.05). Figure 1 presents a case of a patient with corneal ectasia (Fig. 1A) in the right eye and FFKC (Fig. 1B) in the left eye.

### Corneal morphological parameters using the pentacam device and biomechanical parameters using the corvis device

The main morphological and biomechanical parameters are shown in Table 1. Morphological parameters included Pachy (corneal thickness of the thinnest point from the Pentacam HR), ARTh and BAD_D; biomechanical parameters included IR, SP_A1, DA-ratio_2mm, SSI and CBI; and the combined index was the TBI. Statistical analysis showed that there was a significant difference between groups for each parameter, as shown in the table (*P* = 0.000). Bonferroni and post hoc tests indicated that between KC and control eyes, all morphological and biomechanical parameters were significantly different (*P* < 0.05). However, between the FFKC and control eyes, BAD_D, SSI and TBI were not significantly different (*P* = 0.121, *P* = 0.465, *P* = 0.096, respectively), while the other parameters all demonstrated significant differences (*P* < 0.05). Additionally, between the KC and FFKC eyes, the SSI was not significantly different (*P* = 0.132), while the other parameters were all significantly different (*P* < 0.05).

**Table 1:** Morphological and biomechanical parameters in KC eyes, FFKC eyes and control eyes

	KC eyes	FFKC eyes	Control eyes	*p*
Pachy (µm)	467.3 ± 59.1	519.4 ± 28.4	554.1 ± 31.3	0.000^#^
ARTh	199.44 ± 121.01	470.67 ± 109.69	607.65 ± 118.30	0.000^#^
IR (mm^− 1^)	12.20 ± 2.75	8.81 ± 1.14	7.94 ± 0.82	0.000^#^
SP_A1(Hg/mm)	57.69 ± 23.71	87.62 ± 15.45	118.45 ± 14.87	0.000^#^
DA-ratio_2mm	6.47 ± 1.98	4.70 ± 0.63	3.92 ± 0.29	0.000^#^
SSI	0.67 ± 0.13	0.77 ± 0.16	0.83 ± 0.11	0.000^#^
CBI	0.92 ± 0.18	0.48 ± 0.33	0.09 ± 0.08	0.000^&^
BAD_D	11.05 ± 5.16	2.04 ± 0.57	0.89 ± 0.59	0.000^#^
TBI	0.99 ± 0.03	0.67 ± 0.34	0.13 ± 0.14	0.000^&^

KC represents keratoconus; FFKC represents forme frusta keratoconus; Kmax represents the maximum value of corneal curvature;I-S value represents the inferior-superior value; Pachy represents the thinnest corneal thickness; ARTh represents Ambrósio relational thickness horizontal; IR represents integrated radius; SP_A1 represents stiffness parameter at first applanation; DA-ratio_2mm represents deformation amplitude at 2 mm around the center; BAD_D represents Belin-Ambrósio deviation index; CBI represents Corvis biomechanical index; And TBI represents tomographic and biomechanical index. *P* was calculated to determine the difference between the three groups. ^#^ means comparisons of Pachy, ARTh, IR, SP_A1, DA-ratio_2mm, SSI and BAD_D were using ANOVA, and ^&^ means comparisons of CBI and TBI were using Kruskal-Wallis test

For the novel stiffness parameter SSI, the AUC in differentiating eyes with any ectasia from normal eyes was 0.756 (95 % CI 0.625–0.886, *P* = 0.000), with a sensitivity and specificity of 0.899 and 0.609, respectively, when the cut-off value was 0.70.

### ROC curves and the best cut-off points distinguishing abnormal eyes from control eyes

**Fig. 2:**
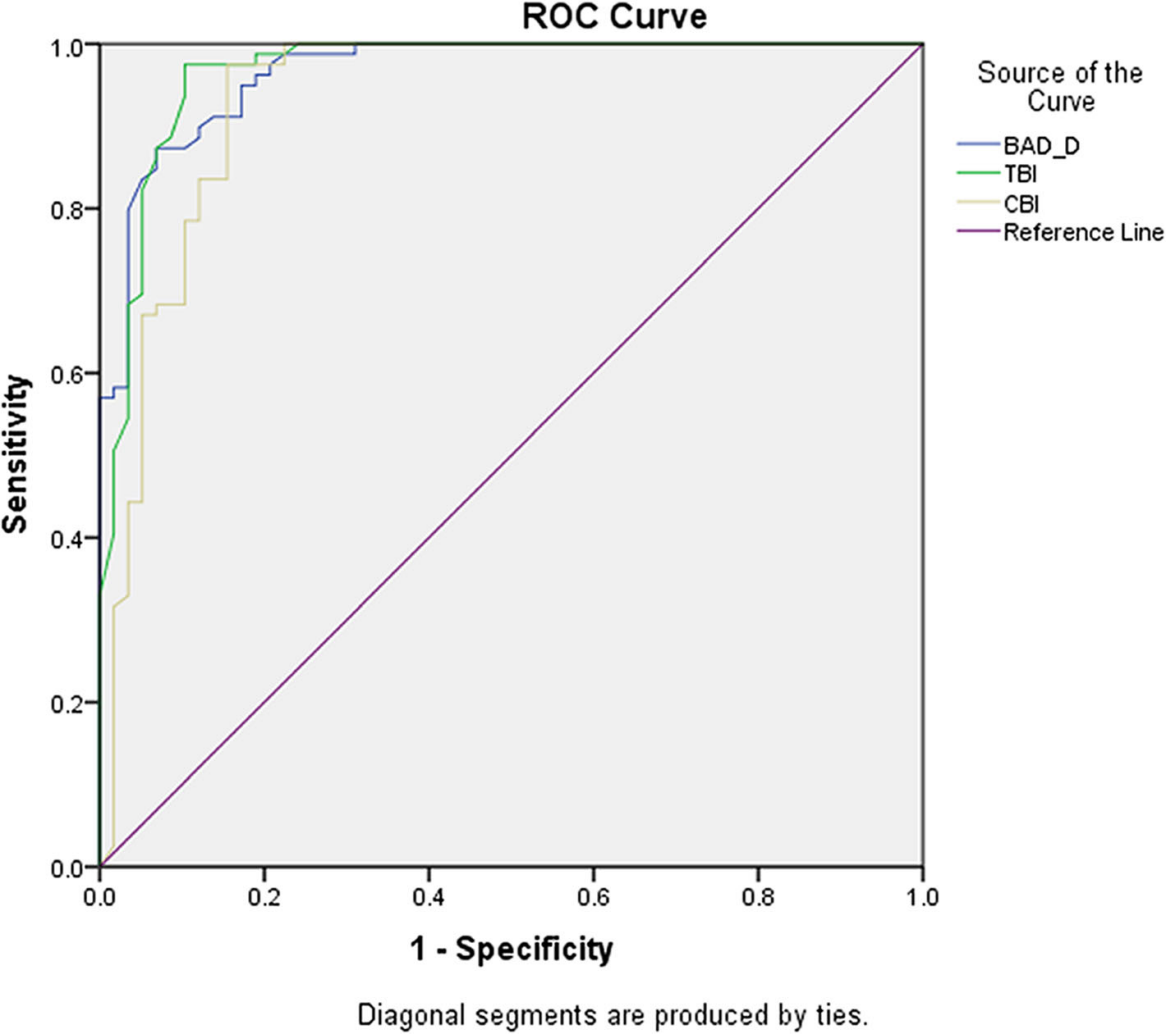
ROC curves of the BAD-D, CBI and TBI combined parameters in distinguishing corneal ectatic eyes (KC and FFKC, *n* = 58) and control eyes (*n* = 79)

To improve the diagnostic efficacy, we chose three combined parameters computed from Pentacam and Corvis parameters and evaluated their receiver operating characteristic (ROC) curves and the best cut-off points [17]. The ROC curves of BAD_D, CBI and TBI for separating abnormal eyes (both KC eyes and FFKC eyes) from control eyes are shown in Fig. 2. The areas under the curve (AUCs), best cut-off points, Youden indices (Youden index = sensitivity + specificity-1), sensitivity and specificity are shown in Table 2. From these results, we found that the TBI had the highest AUC value, reaching 0.966 (95 % CI 0.936 ~ 0.997, *p* = 0.000), followed by BAD_D (0.965, 95 % CI 0.939 ~ 0.992, *p* = 0.000) and CBI (0.934, 95 % CI 0.886 ~ 0.982, *p* = 0.000). The comparison of AUCs showed that although the Youden index of the TBI was highest, the TBI and BAD-D had similar AUCs (0.966 vs. 0.965, DeLong test, *p* = 0.617), while there was a significant difference between the AUCs of the TBI and CBI (0.966 vs. 0.934, *P* = 0.003) and between those of the BAD_D and CBI (0.965 vs. 0.934, *P* = 0.014), indicating a better diagnostic accuracy for the TBI and BAD_D.

**Table 2:** AUC and Best cut-off values of Combined Parameters for Distinguishing any KC Eyes (KC + FFKC, *n* = 58) from Normal Eyes (*n* = 79)

	AUC	Best Cut-off Values	Youden Index	Sensitivity (%)	Specificity (%)
BAD_D	0.965	1.48	0.804	87.3	93.1
CBI	0.934	0.27	0.820	97.5	84.5
TBI	0.966	0.38	0.872	97.5	89.7

AUC represents area under the curve; KC represents keratoconus; FFKC represents forme frusta keratoconus; BAD_D represents Belin-Ambrósio deviation index; CBI represents Corvis biomechanical index; And TBI represents tomographic and biomechanical index

### ROC curves and the best cut-off points distinguishing ffkc eyes from control eyes

**Fig. 3:**
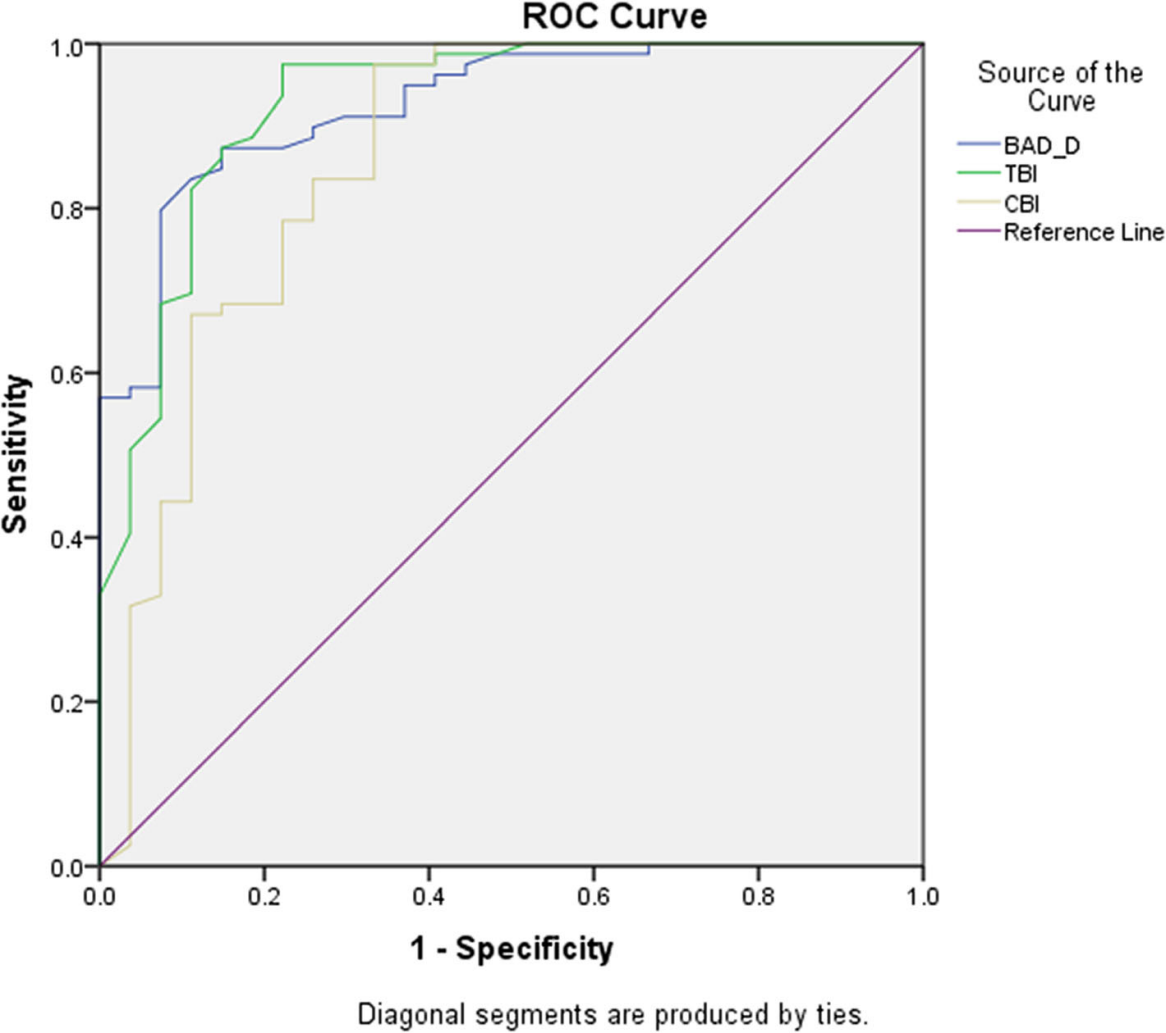
ROC curves of the BAD-D, CBI and TBI combined parameters in distinguishing FFKC (*n* = 27) and control eyes (*n* = 79)

Furthermore, the ROC curves and the best cut-off points were determined to differentiate FFKC from control eyes, as demonstrated in Fig. 3 and Table 3. We found that all 3 parameters had good diagnostic value in detecting FFKC, among which the TBI was the best, with an AUC of 0.928 (*p* = 0.000). In pairwise comparisons, the TBI also had a similar AUC to the BAD_D (0.928 vs. 0.926, *p* = 0.826), and the AUC of the CBI was significantly lower than that of the TBI (0.860 vs. 0.928, *p* = 0.005) and BAD_D (0.860 vs. 0.926, *p* = 0.014).

**Table 3:** AUC and Best cut-off values of Combined Parameters for Distinguishing FFKC (*n* = 27) from Control Eyes (*n* = 79)

	AUC	Best Cut-off Values	Youden Index	Sensitivity (%)	Specificity (%)
BAD_D	0.926	1.48	0.725	87.3	85.2
CBI	0.860	0.27	0.642	97.5	66.7
TBI	0.928	0.38	0.753	97.5	77.8

AUC represents area under the curve; KC represents keratoconus; FFKC represents forme frusta keratoconus; BAD_D represents Belin-Ambrósio deviation index; CBI represents Corvis biomechanical index; And TBI represents tomographic and biomechanical index

### ROC curves and the best cut-off points distinguishing kc eyes from control eyes

The BAD_D, CBI and TBI had excellent performance in detecting frank KC eyes. The AUCs reached 1.000, 1.000 and 0.998, with cut-off values of 2.82, 0.71 and 0.28, respectively. Table 4 shows the ROC curves and the best cut-off points for distinguishing frank KC eyes from control eyes. The CBI, TBI and BAD_D showed no significant difference in the ability to differentiate KC from control eyes (0.998, 1.000 vs. 1.000, *P* > 0.05).

**Table 4:** AUC and Best cut-off values of Combined Parameters for Distinguishing frank KC (*n* = 31) from Control Eyes (*n* = 79)

	AUC	Best Cut-off Values	Youden Index	Sensitivity (%)	Specificity (%)
BAD_D	1.000	2.82	1.000	100.0	100.0
CBI	0.998	0.28	0.975	97.5	100.0
TBI	1.000	0.71	1.000	100.0	100.0

AUC represents area under the curve; KC represents keratoconus; FFKC represents forme frusta keratoconus; BAD_D represents Belin-Ambrósio deviation index; CBI represents Corvis biomechanical index; And TBI represents tomographic and biomechanical index

## Discussion

Keratoconus is a progressive, degenerative disorder that results in thinning and protrusion of the cornea into a conical shape, which causes irreversible vision loss in teenagers and young adults [27]. The cornea is a complex anisotropic composite structure with nonlinear elastic and viscoelastic properties [28]. In eyes with ectasia, specific structural changes occur in the corneal stroma as part of the disease process due to alterations in the viscous and elastic properties of the cornea [27]. Corneal mechanical stability is compromised in keratoconus, sequentially leading to progressive macroscopic morphologic changes [29]. Therefore, evaluation of corneal biomechanical properties is of great value for subclinically ectatic eye screening, and a combination of morphologic and biomechanical examinations theoretically could improve the accuracy of KC diagnoses.

In the current study, we determined the diagnostic ability of Scheimpflug-based tomography combined with biomechanical examination for distinguishing normal eyes from frank KC and FFKC eyes in a Chinese population. Our results indicated that the Scheimpflug-derived morphological and biomechanical examinations were very useful in accurately distinguishing normal from abnormal corneas (including both clinical and potential ectatic eyes). Previous studies using these measuring instruments reported similar outcomes in that the combined parameters were more effective than the individual parameters [24–26, 30]. In this study, we found that the TBI had the highest diagnostic accuracy in detecting corneal ectasia (AUC 0.966, Youden index 0.872), in accordance with many previous studies that found that the TBI was a very accurate and valuable index for detecting ectasia with high sensitivity and specificity in European, Middle East, South American, and even Japanese populations [24–26, 30]. In this study, the TBI at a cut-off value of 0.38 had a sensitivity of 97.5 % with a specificity of 89.7 % in detecting any corneal ectasia, which confirmed its high diagnostic efficacy in Chinese patients. This result was different from that of a previous study in Chinese myopic patients, which found that the CBI was the most sensitive factor in the diagnosis of FFKC eyes (AUC: 0.909, 95 % CI 0.828–0.989), with a very low cut-off value of 0.019 [31]. This divergence might be due to the different inclusion criteria: in that study, the participants were all refractive surgery candidates, which might implicate less severe corneal ectasia.

As illustrated above, FFKC could be considered a potential or early form of keratoconus and might progress into clinical manifestations of KC in the future. Therefore, the ability to distinguish FFKC eyes from normal eyes is of great importance and would be quite valuable in clinical practice. In detecting FFKC eyes, the TBI also had the highest accuracy (AUC 0.928), similar to that of BAD_D (AUC 0.926) and better than that of CBI (AUC 0.860). These results indicate that TBI is superior for discovering mild or subclinical KC. The analyses of VAE patients provided vital information for detecting potential KC eyes at a relatively early stage. The Global Consensus on Keratoconus and Ectatic Diseases in 2015 [6] mentioned that “*real unilateral keratoconus does not exist*”. Accordingly, we think FFKC eyes may already possess some early biomechanical abnormalities despite their relatively normal topographic appearance. In this case, Scheimpflug-derived biomechanical evaluation could help to discover early forms of KC or potential ectasic eyes, which is crucial in refractive surgery screening. In theory, corneal biomechanical property changes might occur before shape changes in subclinical KC [32]. Therefore, biomechanical parameters might be more sensitive to measure than morphological parameters. Interestingly, although the AUC of BAD_D was larger, the sensitivity of CBI was higher than that of BAD_D (97.5 % vs. 87.3 %), which indicated the better screening ability of the biomechanical index. Another point should be noted: the FFKC eyes in this study were not completely tomographically normal, presenting with a BAD_D of 2.04 (although they were all topographical normal). Accordingly, indices containing morphological information such as the TBI and BAD_D were more informative in distinguishing FFKC from normal eyes.

The BAD_D alone was another very useful parameter for detecting corneal ectasia, which is consistent with some other previous studies using only Scheimpflug-based tomography for the early diagnosis of KC [7–10]. The AUC of BAD_D in detecting any KC or FFKC was very close to that of TBI (0.965 vs. 0.966, 0.926 vs. 0.928). The Pentacam alone can detect corneal ectasia with satisfactory efficacy; however, the combination of corneal shape information and biomechanical properties can further improve the diagnostic accuracy, especially the screening sensitivity, which would be of great value in screening any signs of keratoconus before refractive surgery. Considering that the TBI had a higher sensitivity and the BAD_D had better specificity, the TBI may be of higher value for screening subclinical KC, while the BAD_D is more useful for treatment decision-making. Users could choose their preferred index according to different application purposes. The novel in vivo biomechanical parameter SSI was explored in this study. The SSI is a material stiffness parameter that is independent of corneal thickness (CCT) and intraocular pressure (IOP) and significantly correlated only with age [24]. The SSI was set to 1.0 for the average experimental behaviour obtained for corneal tissue at age = 50 years [33]. In this study, we found that for normal eyes of patients in their 20 s, the average SSI was 0.83 ± 0.11, in accordance with previous findings that the SSI is positively correlated with age [24]. Moreover, we found that the SSI decreased in KC eyes, indicating a reduction in corneal stiffness in corneal ectasia. However, the AUC and Youden index of the SSI were not as good as those of the above computed parameters. To our knowledge, there have been few reports of SSI changes in keratoconic eyes and its diagnostic ability in detecting corneal ectasia. Further studies are needed to investigate this issue.

The main limitation of this current study is that it was a cross-sectional diagnostic study. Because of its cross-sectional nature, it was impossible to know how many FFKC corneas would develop to frank KC or how the corneal biomechanical properties would subsequently change. A longitudinal study is needed to illustrate the odds of FFKC eyes with BAD_D, CBI or TBI beyond their cut-off values progressing into frank corneal ectasia. Another limitation is that the number of included cases in this study was relatively small, which could be addressed in a future study including more eligible cases.

## Conclusions

In summary, Scheimpflug-based morphological and biomechanical examination is of great value in detecting and diagnosing KC, especially subclinical KC, in Chinese patients. The TBI is the most accurate parameter, with both a high sensitivity and specificity. The combination of Scheimpflug-based tomography and biomechanical analysis can play an essential role in screening potential corneal ectasia and could maximize LVC surgical safety.

## Data Availability

The datasets used and/or analysed during the current study available from the corresponding author on reasonable request.

## Abbreviations

KC: keratoconus
FFKC: forme frusta keratoconus
BAD_D: Belin-Ambrósio deviation index
CBI: Corvis biomechanical index
TBI: tomographic and biomechanical index
ROC curve: Receiver Operating Characteristics curve
AUC: Area Under Curve
SSI: Stress-strain index
LVC: Laser vision correction
VAEs: very asymmetric eyes
K: Keratometry
Kmax: maximum keratometry
RGP: rigid gas permeable
CCT: central corneal thickness
IOP: intraocular pressure
ARTh: Ambrósio relational thickness to the horizontal profile
I-S: inferior-superior difference value
SPA_1: resultant pressure divided by the deflection amplitude at A1
IR: integrated radius
DA ratio_2: the ratio between deflection amplitude at the apex and the average of the deflection amplitudes at 2 mm
ANOVA: analysis of variance
